# Ascending aortic aneurysm growth in the *Fbln4*^*SMKO*^ mouse is consistent with uniform growth laws

**DOI:** 10.1007/s10237-025-01972-5

**Published:** 2025-07-21

**Authors:** Marisa S. Bazzi, Hadi Wiputra, Weihua Guan, Victor H. Barocas

**Affiliations:** 1 Department of Chemical Engineering and Materials Science, University of Minnesota, Minneapolis, MN 55455 USA; 2 Department of Biomedical Engineering, University of Minnesota, Minneapolis, MN 55455 USA; 3 Division of Biostatistics, School of Public Health, University of Minnesota, Minneapolis, MN 55455 USA

**Keywords:** Cardiovascular, Artery, Growth, Remodeling, Computational

## Abstract

Arterial growth and remodeling (G&R), in response to biomechanical stimuli, plays a pivotal role in vascular health. Disruptions in G&R, often seen in conditions such as aneurysms and atherosclerosis, can lead to pathological changes and pose significant health risks. Assessing risk should not only consider the current state of the aneurysm but also how it develops over the subsequent months. Herein, we make a controlled, subject-specific assessment of maladaptive aortic tissue growth using data previously obtained for the *Fbln4*^*SMKO*^ mouse model. The computational model uses a locally applied continuum G&R approach coupled with fluid–structure interaction (FSI) simulations. Ten mice were studied, exhibiting varying degrees of aneurysm formation over time. This investigation focused on the ascending aorta, where aneurysms develop in the *Fbln4*^*SMKO*^ mouse. A continuous G&R model was tuned and evaluated using information from 2, 4, and 6 months obtained from CT scans. A G&R model with uniform growth laws showed variable accuracy in predicting circumferential growth across different mice, exhibiting both under- and over-estimations compared to in vivo measurements. Modeling prediction showed to be improved by multiple-domain modeling. There is correlation between (1) the fitted circumferential growth time constants and the observed ascending aorta Young’s modulus and (2) the fitted axial growth time constant and the tortuosity index. Furthermore, the ratio of the circumferential growth time constant to the circumferential stress correlated with mouse lifespan more strongly than diameter change, suggesting that analysis of a G&R model may be valuable in predicting risk of aneurysm rupture.

**Supplementary Information:**

The online version contains supplementary material available at 10.1007/s10237-025-01972-5.

## Introduction

Growth and remodeling (G&R) is the process by which living tissues change their physical and mechanical properties in response to their environment. A popular principle in arterial G&R is that the process maintains a homeostatic stress level in the vessel walls (Rodriguez et al. 1994). In healthy arteries, mechanical homeostasis is characterized by a negative feedback loop, where a preferred state is restored after a perturbation. However, when risk factors, such as hypertension and vascular aging, are present, or in arterial disease, such as aneurysm and atherosclerosis, homeostasis can be disrupted, producing a positive feedback loop that promotes pathological G&R of the arterial tissue (Humphrey and Schwartz 2021).

For ascending thoracic aortic aneurysms (ATAA), the positive feedback loop triggers continuous growth and gradual weakening of the vessel wall, increasing the risk of aortic rupture or dissection. Though rupture is rare, it is estimated that more than 50% of the patients with a ruptured ATAA succumb before reaching the hospital, resulting in an overall mortality between 11 and 90% (Raaymakers et al. 1998). A major challenge in ATAA management is our inability to predict which patients are at risk of aneurysm rupture accurately. While aneurysm diameter and growth rate are the measurements guiding surgical decisions, they fall short of providing a comprehensive diagnosis, frequently providing inadequate prediction of the actual risk (Coady et al. 1999).

Additional metrics beyond growth and maximum diameter must be considered to advance diagnostic accuracy and improve the prediction of rupture risk. Several studies have investigated alternative biomarkers, such as blood flow dynamics, mechanical properties, and aortic tortuosity as potential additional indicators predicting adverse outcomes in patients with aneurysms (Urbonavicius et al. 2008; Pappu et al. 2008; Bazzi et al. 2020; Signorelli et al. 2018). However, those metrics often evaluate a snapshot rather than accounting for the time-dependence nature of the diseases.

In this study, we investigated the idea that progressive changes due to maladaptive aortic G&R can be interpreted through the lens of a computational G&R model. The *Fbln4*^*SMKO*^ mouse (Wagenseil et al. 2005) provided an ideal system for this study. The genetic and lifestyle reproducibility of mouse studies, combined with its short lifespan, yield a much better-controlled system than the human. In addition, a completed and detailed longitudinal study of aortic G&R in the *Fbln4*^*SMKO*^ mouse, including CT imaging and postmortem mechanical testing on each mouse, has already been performed (Bazzi et al. 2022). Herein, we used a simplified growth and remodeling model rooted in a continuum approach, combined with comprehensive fluid–solid interaction (FSI) simulations tailored to individual mice, to test two hypotheses:

1. Even a relatively simple G&R model can be regressed to longitudinal aortic geometry data and used to predict future changes in aortic shape.
2. The fitted growth parameters correlate with the ATAA outcome.

Reconstructed 3D geometric models were used in conjunction with the scans at 2 and 4 months to tune the G&R model, which was then used to predict 6-month geometry for model assessment/validation. To address the second hypothesis, the fitted G&R model parameters were correlated with the lifespan of the mouse along with various other measures of aortic health.

## Methods

**Fig. 1:**
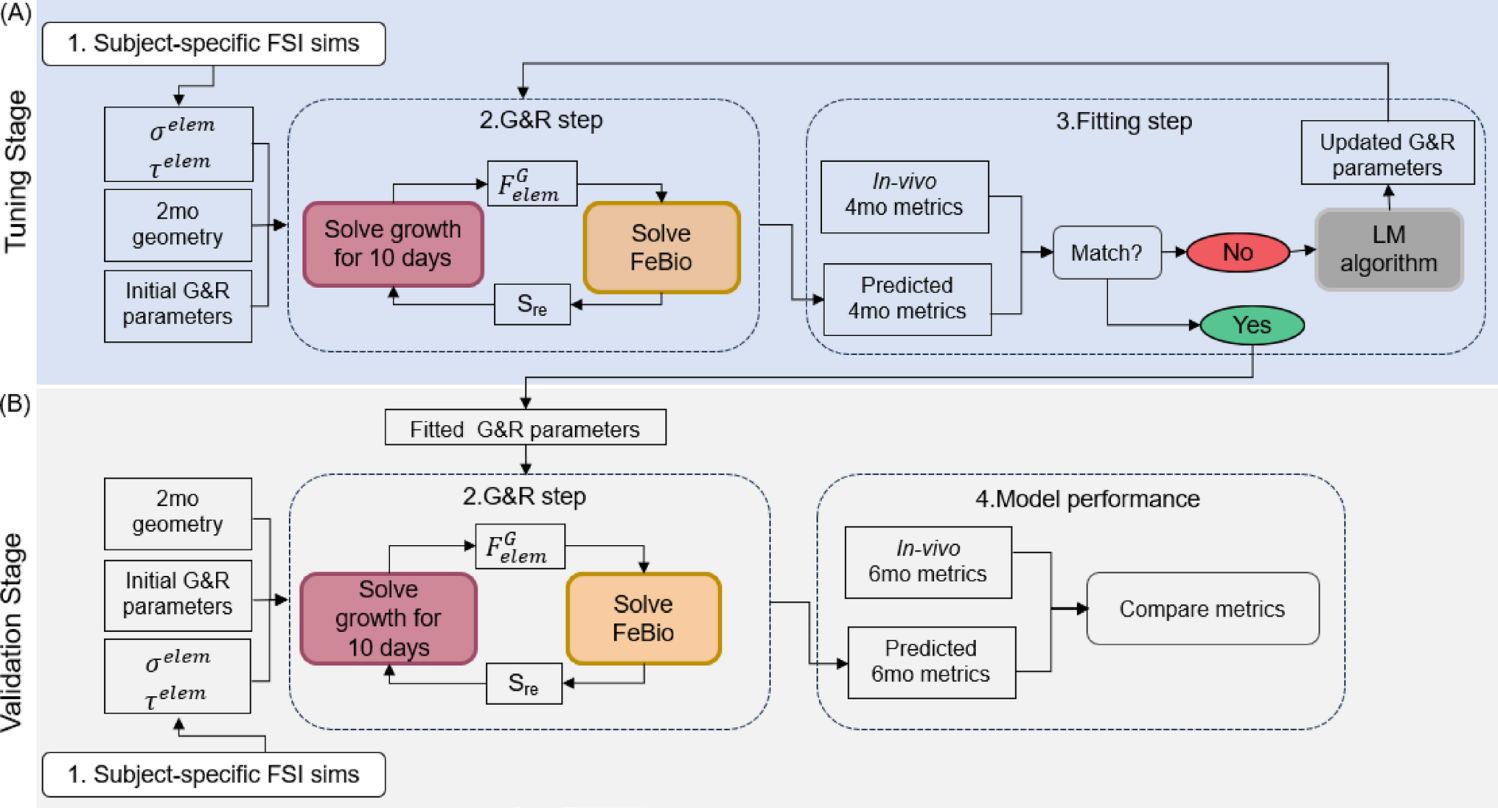
Workflow of subject-specific hemodynamics-driven growth and remodeling model. **A** Steps for the tuning stage using data from 2- and 4-month mouse models. **B** Steps for the validation stage using the fitted parameters from the tuning stage to evaluate the model performance against the 6-month in vivo metrics

In this study, we brought together FSI and G&R models, as shown in Fig. 1. The computational framework is split into tuning and validation stages. For the tuning stage, information for 2 and 4 months is used to fit the growth parameters in the computational model. Figure 1A shows the workflow for the tuning stages, whose steps are as follows:

1. Subject-specific FSI simulation (details in Sect. 2.3–2.4): An unsteady blood flow simulation was solved for the subject-specific geometry (age 2 months) using the svFSI solver from SimVascular (Updegrove et al. 2017). The time-averaged wall stresses were extracted from each element and used as input for the growth and remodeling (G&R) model.
2. G&R model (details in Sects. 2.5–2.7): The G&R model was solved for each element in the ascending aortic region using a time step of 10 days of simulated tissue growth. After 10 days, the local growth tensor was extracted and used as input for FEBio to solve for the equilibrium geometry of the ascending aorta, from which we extracted residual stress (*S*_re_) and updated the total stress in the G&R model. More details about the G&R model can be found in Sects. 2.5 and 2.6.
3. Fitting parameters (details in Sects. 2.8–2.9): After 60 days of simulated remodeling, the predicted geometry was calculated from the G&R model and compared to the axial and circumferential geometry measured in vivo at 4 months. If the predicted measurements had not yet converged to a minimum error with respect to in vivo measurements, a new set of G&R parameters was calculated using the Levenberg-Marquardt algorithm (Gavin 2019), and the process restarted at step 2.

For the validation stage, shown in Fig. 1B, information for two- and six-month mice was used to assess the model performance. In this stage, the G&R model was applied for 120 days of simulated time using the fitted G&R parameters obtained from the tuning stage. The predicted metrics were extracted from the final geometry and compared to the in vivo metrics from the 6-month-old mice.

### Mouse model

The study was performed using data previously published for ten mice lacking expression of fibulin-4 in smooth muscle cells (*Fbln4*^*SMKO*^) (Bazzi et al. 2022). Fibulin-4 is critical for elastic fiber assembly, and *Fbln4*^*SMKO*^ mice have ATAAs with about 50% penetrance. Mice were monitored regularly; deceased mice were immediately collected, and the thoracic aorta was removed for mechanical testing. Any mice still alive at 25 months of age were euthanized by CO_2_ inhalation, and the thoracic aorta was removed for mechanical testing, as described below. More details about the mouse study can be found in our previous paper (Bazzi et al. 2022).

### Region of interest

**Fig. 2:**
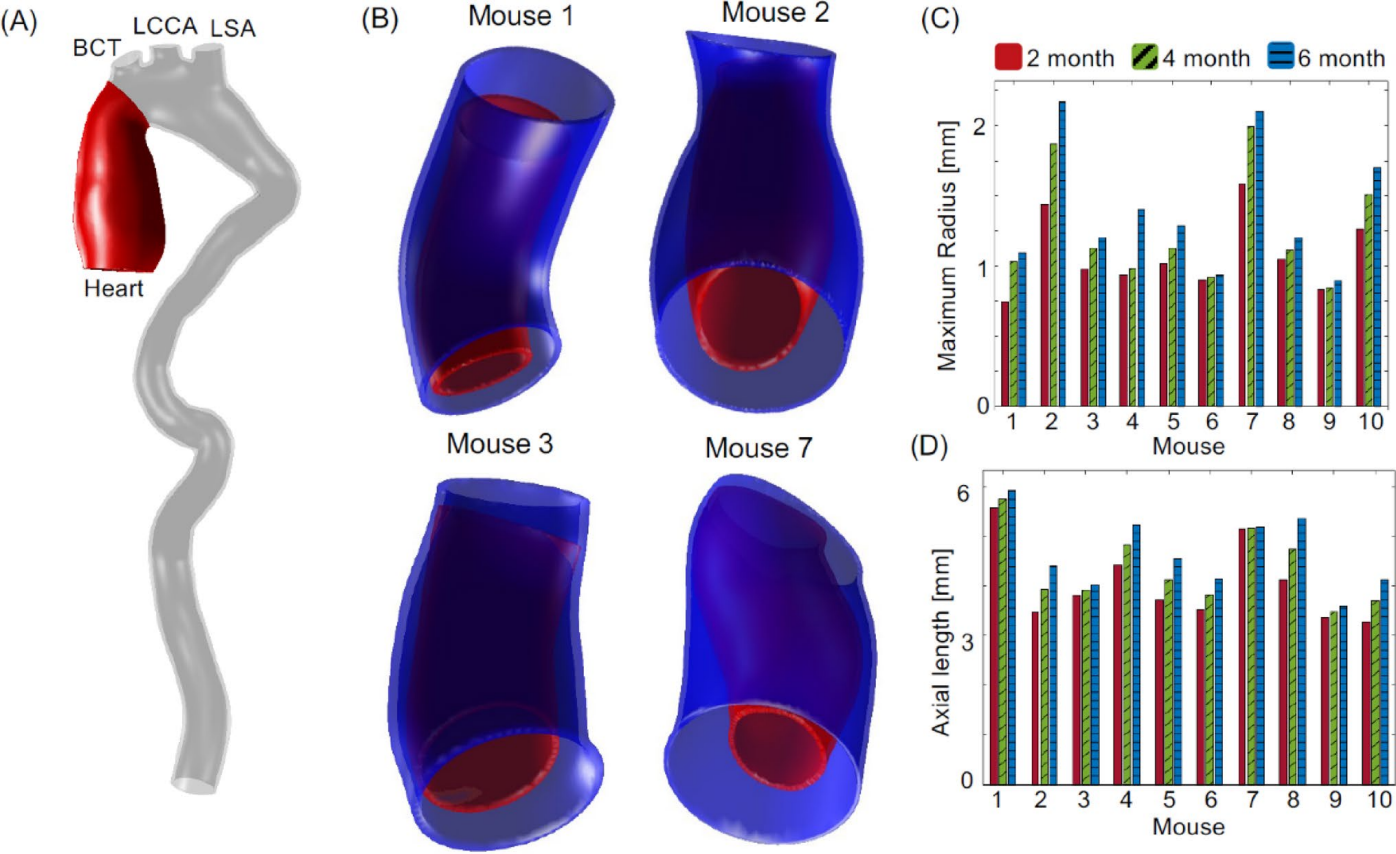
**A** Region of interested (in red) delimited by the heart and brachiocephalic trunk (BCT). **B** Geometries of ascending thoracic aorta (ATA) for 2 (red) and 6 months (blue) of four different mice models. **C** Comparison between maximum ATA diameter at 2, 4, and 6 months for each mouse. **D** ATA axial length at 2, 4, and 6 months for each mouse. Red bars are 2-month, green bars are 4-month, and blue bars are 6-month measurements

Although the FSI simulations were performed on the entire thoracic aorta, the analysis focuses on the ascending thoracic aorta, where aneurysms are formed for *Fbln4*^*SMKO*^ mouse models. Figure 2A shows the 3D ascending aortic geometry for four mice selected to represent the ten used in this study. The different mice show a considerable variation in circumferential growth, with mice 2 and 7 exhibiting large aneurysm formation, mouse 3 a moderate aneurysm, and mouse 1 a mild aneurysm. In terms of temporal change in the diameter, mice 2 and 7 had a 34% and 33% increase in the ascending thoracic artery mean radius from 2 to 6 months, respectively, mouse 3 had 28% increase, and mouse model 1 had a 20% increase.

### Fluid–solid interaction simulations

We segmented mouse CT images taken at mid-diastole as described in our previous work (Bazzi et al. 2022). Two main landmarks—the heart in the ascending region and the celiac artery in the descending region, were used to ensure the selection of the same portion of the aorta from the medical images. The brachiocephalic trunk (BCT), left common carotid artery (LCCA), and left subclavian artery (LSA) were also partially segmented and used as part of boundary conditions for the blood flow simulations. The image segmentation, model generation, and 3D meshing were performed in SimVascular (Updegrove et al. 2017) with Meshmixer (Autodesk, Inc.) for supplemental editing.

The FSI simulation requires two separate conforming meshes: a mesh for the fluid domain, and a mesh for the structural domain. For the structural domain, a mouse-specific unloaded wall thickness was imposed based on experimental measurement for the ascending aortic region and was treated as constant over the length of the aorta.

**Fig. 3:**
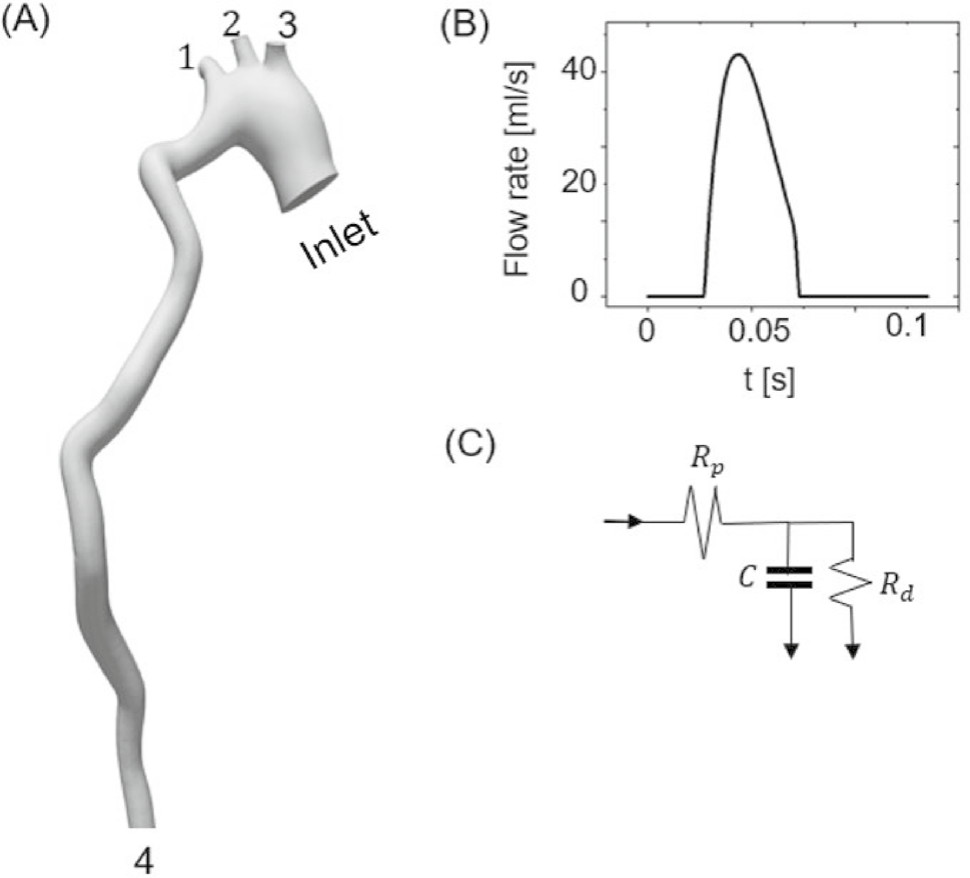
**A** Fluid domain geometry with inlet and four outflow boundaries labeled. **B** Flow rate waveform for inlet. **C** Three-element RCR model for outlets at locations 1–4. RCR model parameters are given in Table 1

For the fluid domain (Fig. 3A), a parabolic waveform from the mouse study of Cuomo et al. (2017) was used as the inlet boundary condition, as shown in Fig. 3B. For each outflow boundary condition, a three-element RCR (Resistor–Capacitor–Resistor) circuit analog model was prescribed, as shown in Fig. 3C. The value for the distal resistance (*R*_d_), proximal resistance (*R*_p_) and compliance (*C*) were based on literature values (Cuomo et al. 2015) and tuned for an average aortic pressure of about 90 mmHg. The flow boundary conditions were assumed to be the same for all mice. Values of the fitted RCRs are shown in Table 1. The blood viscosity was described using the non-Newtonian Carreau-Yasuda model to account for shear thinning of the blood in the moderate Reynolds number present in the mouse aorta (order of 200) (Huo et al. 2008), as described previously (Bazzi et al. 2022).

**Table 1:** Outflow model parameter values

	*R*_p_ (Pa·s/mm^3^)	*C* (Pa/mm^3^)	*R*_d_ (Pa·s/mm^3^)
1. Left subclavian artery	$19.58$	$5.5\times {10}^{-4}$	$286.2$
2. Left common carotid	$44.70$	$3.23\times {10}^{-4}$	$488.0$
3. Brachiocephalic trunk	$21.55$	$3.54\times {10}^{-4}$	$443.2$
4. Outlet (abdominal aorta)	$10.30$	$5.41\times {10}^{-4}$	$443.2$

A Robin-type boundary condition was used to account for the viscoelastic support of the outer arterial wall (Bäumler et al. 2020), given by:

1 $${\varvec{\sigma}}_{{\varvec{s}}} \cdot {\varvec{n}} = - k_{s} {\varvec{u}} - c_{s} \frac{{\partial {\varvec{u}}}}{\partial t} - p_{0} {\varvec{n}}$$

where ${k}_{s}$ and ${c}_{s}$ are viscoelastic parameters that modulate the external tissue response, ${p}_{0}$ is the external pressure in the thoracic and abdominal cavity, ${\varvec{u}}$ is the displacement field, ${{\varvec{\sigma}}}_{{\varvec{s}}}$ is the Cauchy stress tensor in the structural domain, and ${\varvec{n}}$ is unit the normal vector for the outer wall surface. We prescribed $k_{s} = 1 \times 10^{7} \;{\text{N}}\;{\text{s}}\;{\text{ m}}^{ - 3}$ and set ${p}_{0}={c}_{s}=0$, which is within the range of parameters reported in Bäumler et al. (2020).

To account for the mechanical load on the structural domain from the blood pressure at the mid-diastole geometry used, we prestressed the initial geometry using the approach proposed by Hsu and Bazilevs (2011), using in-built prestressing routine in svFSI solver of SimVascular (Vedula et al.).

### Solid domain constitutive model

The solid domain was modeled as a homogenous, isotropic, nonlinear hyperelastic material, described by the Neo Hookean model, where the strain energy density is written as

2 $${W}_{NH}=\frac{\mu }{2}\left({I}_{1}-3\right)-\mu \text{ln}\left(J\right)+\frac{\lambda }{2}{\left[\text{ln}\left(J\right)\right]}^{2}$$

where ${I}_{1}$ is the first invariant of the right Cauchy-Green deformation tensor ***C***. $J$ is the determinant of elastic deformation tensor ${{\varvec{F}}}^{e}$, $\lambda$ is the first Lamé parameter, and $\mu$ is the shear modulus or the second Lamé parameter. The Lamé parameters can be written as functions of the elastic modulus, *E* and bulk modulus, $\nu$

3 $$\mu =\frac{E}{2\left(1+\nu \right)}$$

4 $$\lambda =\frac{\nu E}{(1+\nu )(1-2\nu )}$$

For the simulation, the values of *E* were subject-specific and based on postmortem measurements of the ascending aorta, as given in Table 2. The Poisson’s ratio $\nu$ was set to 0.44 (Nolan and McGarry 2016).

**Table 2:** Sex, lifespan, aortic tortuosity index (ATI) and postmortem measurements of ascending aortic elastic modulus *E*_desc_ and descending aortic modulus *E*_desc_

Mouse	Sex	Lifespan (months)	*E*_asc_ (kPa)	*E*_desc_ (kPa)	ATI
1	Male	17.87	2234	700	45.34
2	Male	18.2	1164	719	59.53
3	Male	22.37	1222	1094	37.87
4	Male	13.27	464	418	52.03
5	Female	21.93	881	867	53.57
6	Female	25*	1595	781	39.86
7	Female	12.37	113	748	35.44
8	Female	25*	930	961	38.78
9	Female	25*	644	631	22.8
10	Male	25*	1033	684	28.74

*Killed at 25 months

### Growth law

Tissue growth in the aortic wall was modeled as anisotropic kinematic growth in response to chronic changes in the hemodynamics. The combination of an anisotropic growth model with an isotropic mechanical model may seem counterintuitive, but it has been successful in other growth and remodeling (Taber 2009, 2020) and is used here as a simple starting point. The hemodynamics-based stress was calculated using the svFSI solver from SimVascular (Updegrove et al. 2017). The stresses were then extracted for each mesh element and used to determine the local growth and remodeling. The growth laws are based on Alford and Taber (2008):

5 $$\frac{\partial {\lambda }_{gr}^{\text{elem}}}{\partial t}=\frac{1}{{T}_{r}}\left(\frac{{\sigma }_{\theta \theta }^{\text{elem}}}{{\widehat{\sigma }}_{\theta \theta }}-1\right){\lambda }_{gr}^{\text{elem}}$$

6 $$\frac{\partial {\lambda }_{g\theta }^{\text{elem}}}{\partial t}=\left[\frac{1}{{T}_{\theta }}\left(\frac{{\sigma }_{\theta \theta }^{\text{elem}}}{{\widehat{\sigma }}_{\theta \theta }}-1\right)+\frac{1}{{T}_{\tau }}\left(\frac{{\tau }_{w}^{\text{elem}}}{{\widehat{\tau }}_{w}}-1\right)\right]{\lambda }_{g\theta }^{i}$$

7 $$\frac{\partial {\lambda }_{gs}^{\text{elem}}}{\partial t}=\frac{1}{{T}_{s}}\left(\frac{{\sigma }_{ss}^{\text{elem}}}{{\widehat{\sigma }}_{ss}}-1\right){\lambda }_{gs}^{\text{elem}}$$

where ${T}_{r}$, ${T}_{\theta }$, ${T}_{\tau }$, and ${T}_{s}$ are time constants, and hats (^) indicates homeostatic target stress values. ${\lambda }_{gr}^{\text{elem}}$, ${\lambda }_{g\theta }^{\text{elem}},$ and ${\lambda }_{gs}^{\text{elem}}$ are radial (*r*), circumferential ($\theta$), and axial (*s*) growth stretch ratios, respectively, for each element $elem$. The stresses ${\sigma }_{\theta \theta }^{\text{elem}}$, ${\sigma }_{ss}^{\text{elem}}$, and ${\tau }_{w}^{\text{elem}}$ are the circumferential Cauchy stress, axial Cauchy stress, and wall shear stress for each element $elem$, as calculated by the full FSI simulation. For G&R equilibrium, ${\sigma }_{\theta \theta }={\widehat{\sigma }}_{\theta \theta }$, ${\tau }_{w}={\widehat{\tau }}_{w}$, and ${\sigma }_{ss}={\widehat{\sigma }}_{ss}$ for all elements in the artery.

Based on Eqs. (5)–(7), the growth tensor ${{\varvec{G}}}^{\text{elem}}$ was defined for each element in the mesh. It is written in the principal coordinate system as:

8 $${{\varvec{G}}}^{\text{elem}}=\left[\begin{array}{ccc}{\lambda }_{gr}^{\text{elem}}& 0& 0\\ 0& {\lambda }_{g\theta }^{\text{elem}}& 0\\ 0& 0& {\lambda }_{gz}^{\text{elem}}\end{array}\right]$$

The total deformation ${\varvec{F}}$ experienced by the tissue relative to its initial state at any given time is:

9 $${\varvec{F}}={{\varvec{F}}}^{e}\cdot {\varvec{G}}$$

where ${{\varvec{F}}}^{e}$ elastic deformation tensor is due to external loads

### Solving the G&R problem

In the G&R solution, to enhance simplicity and computational efficiency, the domain was restricted to the ascending aortic region, specifically between the heart and the brachiocephalic trunk. This region was selected as it is where the aneurysm was developing. The G&R problem was solved by the finite-element method, using the prestrain framework (Maas et al. 2016) in FEBio2.9.1 (Maas et al. 2012). Each element of the FE mesh was prescribed as a prestrained elastic material to solve for stress equilibrium arising from uneven rates of growth at different regions of the geometry. Consistency was maintained by utilizing the same material model and initial properties as the prior fluid–structure interaction (FSI) simulation, ensuring a seamless transition between computational platforms.

Boundary conditions were established by imposing displacements at both the inlet and outlet, which for the G&R model is defined as the region close to the brachycephalic trunk. These displacements were calculated through linear interpolation between the 2-month and 6-month time points.

### Mapping meshes from 2 to 6 months

**Fig. 4:**
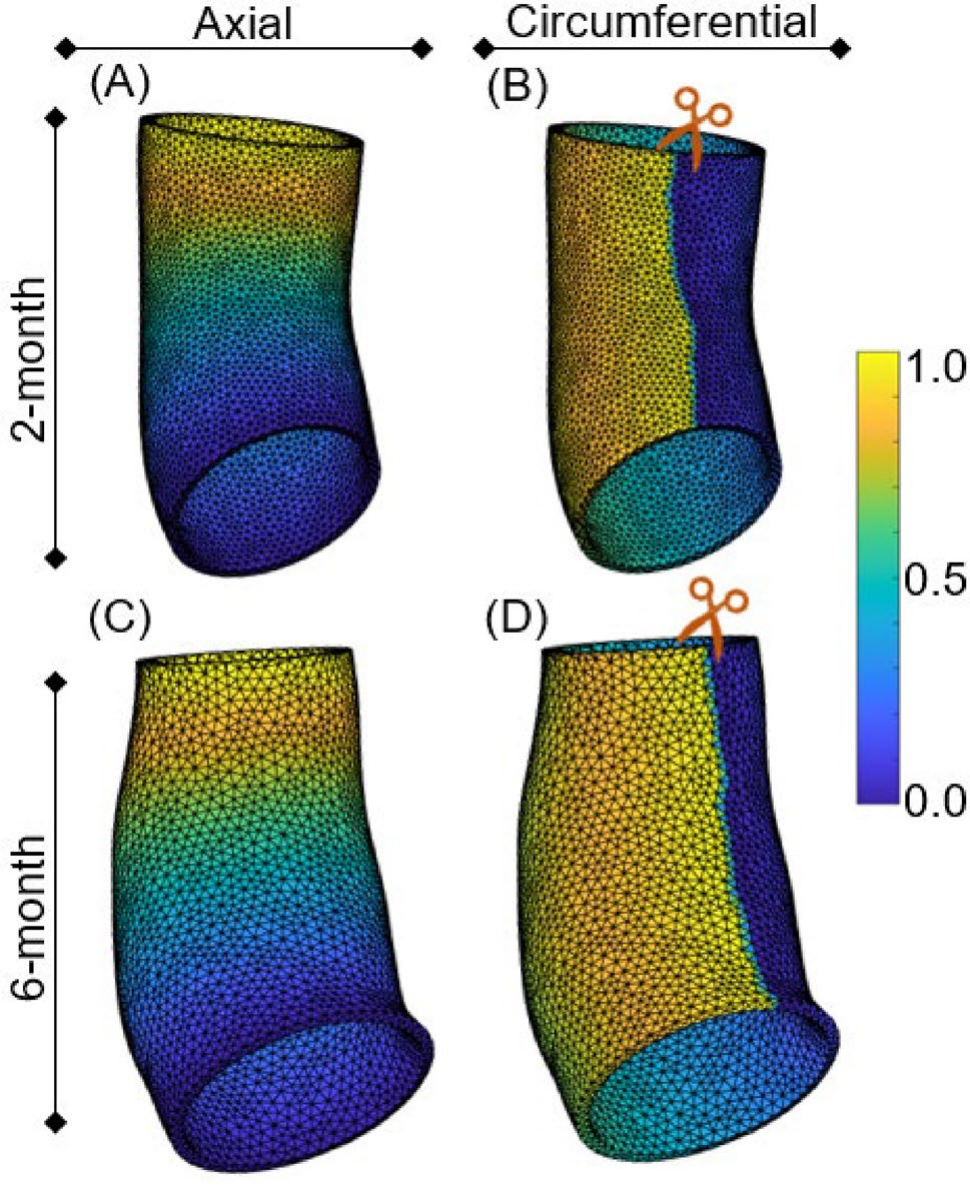
Heat-transfer-based mapping solves the steady-state heat transfer equation for 2-month (**A** and **B**) and 6-month (**C** and **D**) geometry

Since the meshing was performed independently for each age, it was necessary to ensure a one-to-one mapping between the time points for validation purposes, we used a diffusion-based technique (Korenczuk et al. 2019) to define a coordinate system and map between geometries. In this approach, we solve elliptic problem ${\nabla }^{2}T=0$ twice, once to determine the axial mapping and once to determine the circumferential mapping, for each geometric model. For the axial (*s*) mapping, the inlet temperature was set be $T=0$, and the outlet $T=1$. The solution shown in Fig. 4A and C provided a smooth function that varied from 0 to 1 along the length of the domain. For the circumferential $(\theta )$ mapping, the mesh was cut throughout the axial direction along the shortest geodesic distance that connected the inlet to the outlet. The boundary conditions of the problem were $T=0$ on one surface of the cut, and $T=1$ in the other surface; this solution resulted in a smooth circumferential mapping around the vessel surface, as shown in Fig. 4B and D. Since the wall thickness was assumed constant, a simpler linear mapping through the thickness was sufficient to define the radial position.

### Fitting G&R parameters

**Fig. 5:**
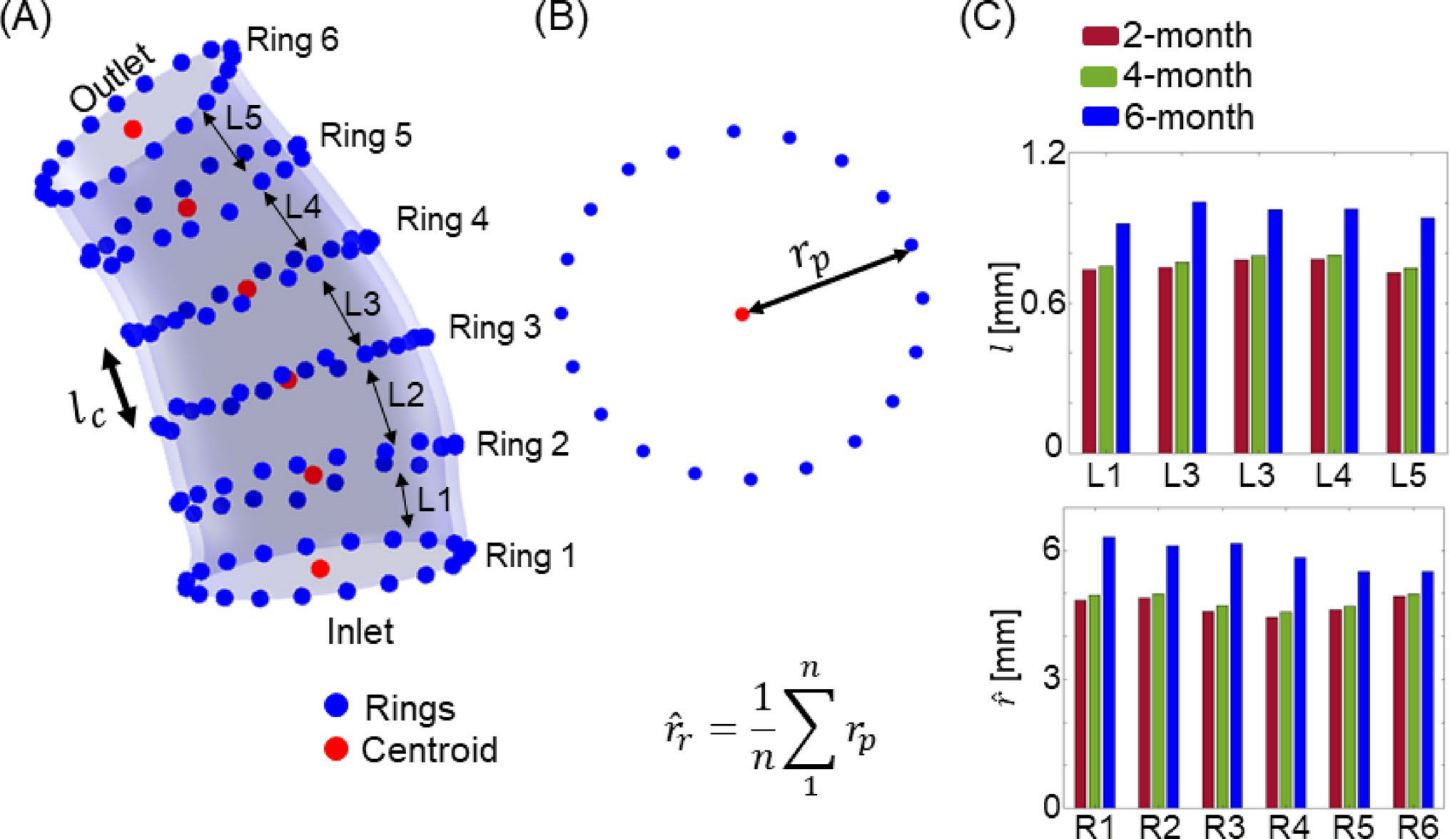
**A** Ascending aorta (transparent blue) with selected nodes forming 6 equally spaced rings along the geometry, from which we calculated lengthening ($${L}_{c}$$, c=1 to 5) from the distance between two consecutives rings. **B** The radius $$({\widehat{r}}_{r})$$ is calculated as the average of the distance between each node and the centroid in the ring. **C** Axial length ($$l)$$ and average radius *(*$$\widehat{r})$$ for each ring for mouse #1

The time constants associated with circumferential $${(T}_{\theta })$$ and axial ($${T}_{s})$$ growth were used as fitting parameters. The choice was based on our ability to calculate both change in radius, and axial growth from the images. Since we had no information about changes in the thickness, we kept the time constant for radial growth $${(T}_{r})$$ fixed. Values for the target stresses and radial growth constant are based on literature values (Alford and Taber 2008). As a result, the model had many parameters but only two that were varied for fitting (Fig. 5).

For the same reason, changes in thickness were not used, only measurements of circumferential and axial growth were used for the optimization of the growth parameters. For circumferential growth, the axial and circumferential mapping were used to create equally spaced rings from the heart to BCT as shown in Fig. 5. The radius (${\widehat{r}}_{r})$ for each ring *r* was calculated by taking the mean distance between each point in the ring to the centroid, as shown in Fig. 5B. For axial lengthening $({L}_{c}$), the distance between two consecutive centroids was calculated.

The cost function (as defined by the chi-square error criterion, ${\upchi }_{r}^{2}$) was minimized using the Levenberg–Marquardt damped least-square algorithm (Gavin 2019). The cost function was:

10 $${\upchi }_{r}^{2} = \mathop \sum \limits_{i = 1}^{{2{\text{n}} - 1{ }}} \left[ {\frac{{X_{r}^{{4{\text{mo}}_{{\text{in-vivo}}} }} - {\text{X}}_{{\text{r}}}^{{4{\text{mo}}_{{{\text{num}}}} }} \left( {\varvec{p}} \right)}}{\sigma }} \right]^{2}$$

where $X_{r}^{{4{\text{mo}}_{{\text{in-vivo}}} }}$ is the 4-month in vivo growth for each ring *r*, $X_{r}^{{4{\text{mo}}_{{{\text{num}}}} }}$ is the 4-month growth calculated using the G&R model, ${\varvec{p}}$ is the parameter vector, and *σ* is the measurement error covariance.

### Multiple-domain segmentation

**Fig. 6:**
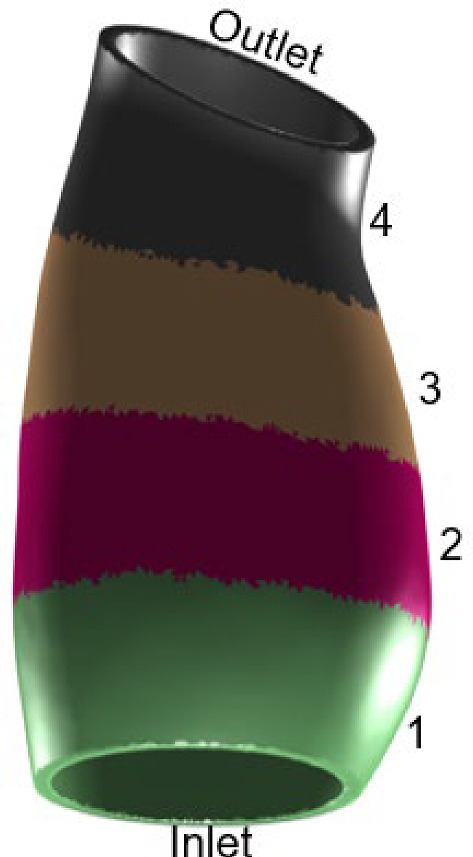
Four-domain segmentation, $k=1 to 4$, along the centerline

The problem was repeated with spatially distributed heterogeneous growth parameters. The 3D geometries were segmented into four equally spaced domains along the centerline as shown in Fig. 6. For each domain k, separate time constants ${T}_{\theta }^{k}$ and ${T}_{s}^{k}$ were assigned.

### Performance analysis

While the cost function for optimization was based on the chi-square error criterion given in Eq. (10), the performance analysis for the validation step was calculated using the relative difference for radius, $\Delta r$ and relative difference for axial lengthening, $\Delta l$, normalizing by the experimentally measured values.

11 $$\Delta r=\frac{{r}^{p }-{r}^{m}}{{r}^{m}}$$

12 $$\Delta l=\frac{{l}^{p }-{l}^{m}}{{l}^{m}}$$

where $r$ stands for radius and $l$ for axial length, as defined in Fig. 5. The superscript m (“measured”) stands for measures coming from medical images, and the superscript p (“predicted”) stands for measures obtained from the predicted geometry.

We specifically chose 4 rings along the geometry, Ring 2 through Ring 5, as shown in Fig. 5A for the calculation of validation performance with Eq. (11). Ring 1 and Ring 6 (i.e., the inlet and outlet rings) were excluded from the analysis to avoid bias from the boundary conditions.

Similarly, the performance of the single-domain growth model vis-à-vis axial lengthening was done using Eq. (12). The total axial length was defined as the sum of the distances between adjacent centroids (see Fig. 5). Unlike the radius change analysis, the analysis of axial length change included the entire vessel from the inlet to the outlet.

### Correlation analysis

The correlation between the fitted radial and axial growth constant and various experimentally measured quantities was calculated. Correlations were performed on a few parameters:

1. Lifespan (LS)—as an indicator of the aneurysm outcome
2. Maximum diameter at 6 months (${D}_{6\text{mo}}^{\text{Max}}$)
3. Change in maximum aortic diameter from 2 to 6 months $\left( {\Delta {\text{D}}} \right)$
4. Aortic tortuosity index at 6 months (ATI)
5. Postmortem measurements of ascending (*E*_asc_) and descending (*E*_des_) aorta’s Young’s modulus.
6. Circumferential (${T}_{\uptheta }$) and axial (${T}_{\text{s}}$) growth parameters.
7. Ratio between the circumferential (${T}_{\uptheta }$) growth parameter and the circumferential target stress (${\overline{\overline{\sigma }}}_{\theta \theta }$).

Out of the group of ten mice, four were killed at the age of 25 months, leading to data being capped at this maximum limit. To accommodate this censored data, we employed the Tobit model (Tobin 1985), which is expressed as:

13 $$\begin{aligned} & Y_{t} = X_{t} \beta + u_{t} \quad {\text{if }} X_{t} \beta + u_{t} < Y_{{{\text{max}}}} \\ & Y_{t} = Y_{{{\text{max}}}} \quad \quad \;\;\; {\text{if}} X_{t} \beta + u_{t} \ge Y_{{{\text{max}}}} \\ \end{aligned}$$

In this model ${Y}_{t}$ is the dependent variable, which in our study is the lifespan of the mouse; ${X}_{t}$ stands for the independent variables; β is the fitting coefficient; and ${Y}_{\text{max}}$ is the censor cutoff, 25 months in our case. The stochastic error ${u}_{t}$ is assumed to be normally distributed with mean zero and a constant variance σ^2^. Here, the subscript t ranges from 1 to N, where N represents the total number of observations in our dataset.

Due to the Tobit model's restrictions on the dependent variable, the conventional R-squared is not a suitable metric for assessing goodness of fit. Instead, we used the modified McKelvey and Zavoina pseudo-R-squared (McKelvey and Zavoina 1975), as it has demonstrated an ability to restore standard R-squared values typically applicable to non-censored data.

14 $${r}^{2}=\frac{\sum_{i=1}^{N}{\left({Y}_{t}-\overline{{Y }_{t}}\right)}^{2}}{\sum_{i=1}^{N}{\left({Y}_{t}-\overline{{Y }_{t}}\right)}^{2}+N{\sigma }^{2}}$$

where $\overline{{Y }_{t}}$ is the mean of $Y$.

## Results

### Single-domain model: detailed analysis of a representative case

This initial part of the results focuses on the single-domain model and its performance in both the tuning and validation stages. To begin, we examine a specific mouse, mouse #2, which was selected due to its development of severe aneurysm, showcasing significant growth from the 2-month to 6-month mark, almost doubling its maximum diameter.

**Fig. 7:**
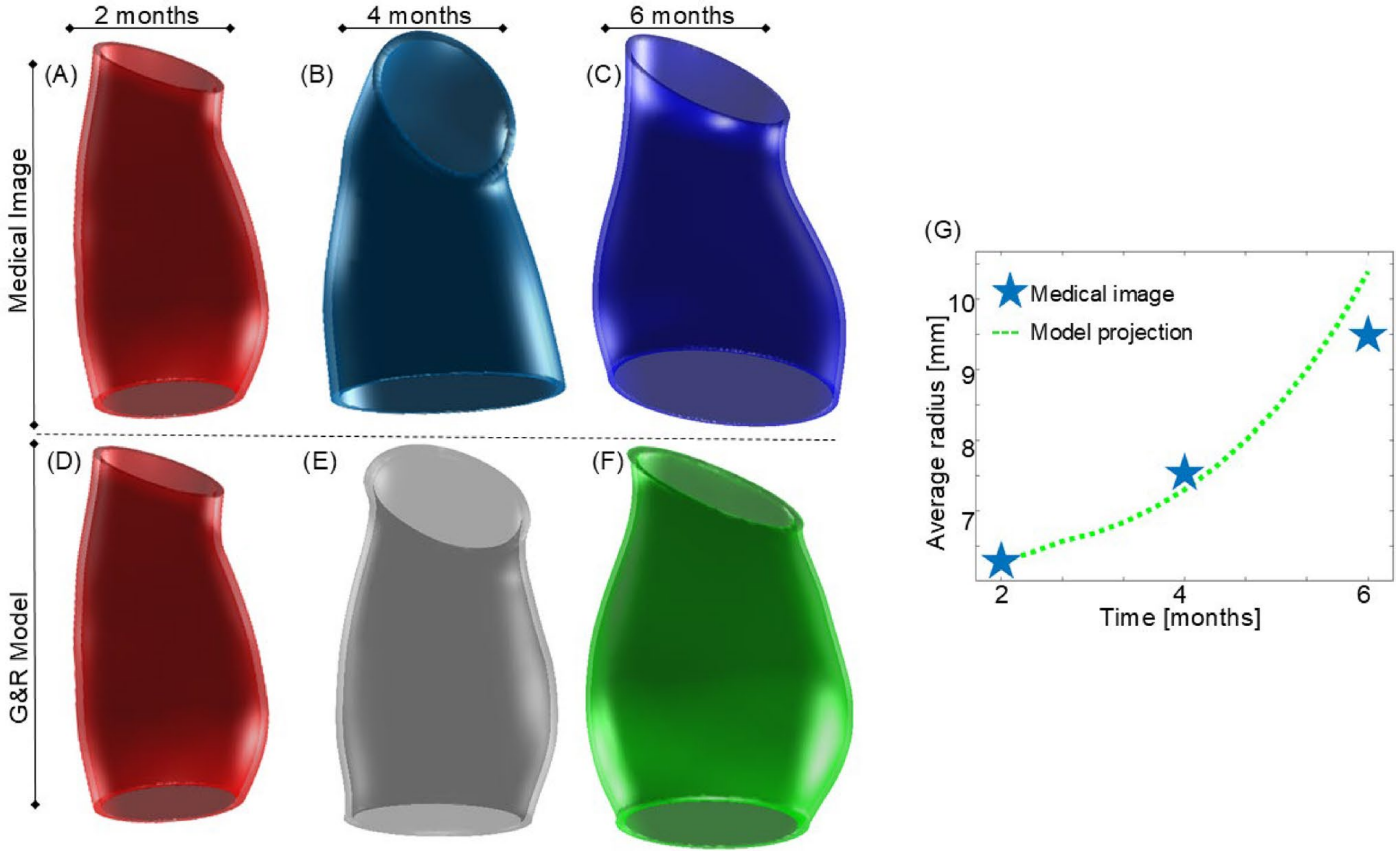
In vivo geometries capture at **A** 2 , **B** 4, and **C** 6 months of the ascending aortic region obtained via medical image versus predicted geometries at **D** 2, **B** 4, and **C** 6 months obtained from the growth model. **G** Time evolution of the average radius of the model (green) compared to in vivo measurements (blue)

Figure 7A–C presents a comparison between the geometries obtained from medical images at 2, 4, and 6 months, respectively, versus geometries predicted by the growth model Fig. 7D,E. Figure 7G displays the temporal evolution of the average radius of predicted geometry (green) compared to the in vivo measurements (blue). We can observe that the model slightly underfits the average radius at 4mo and overestimates it at 6mo.

### Single domain model: assessment of performance on radial expansion across subjects

**Fig. 8:**
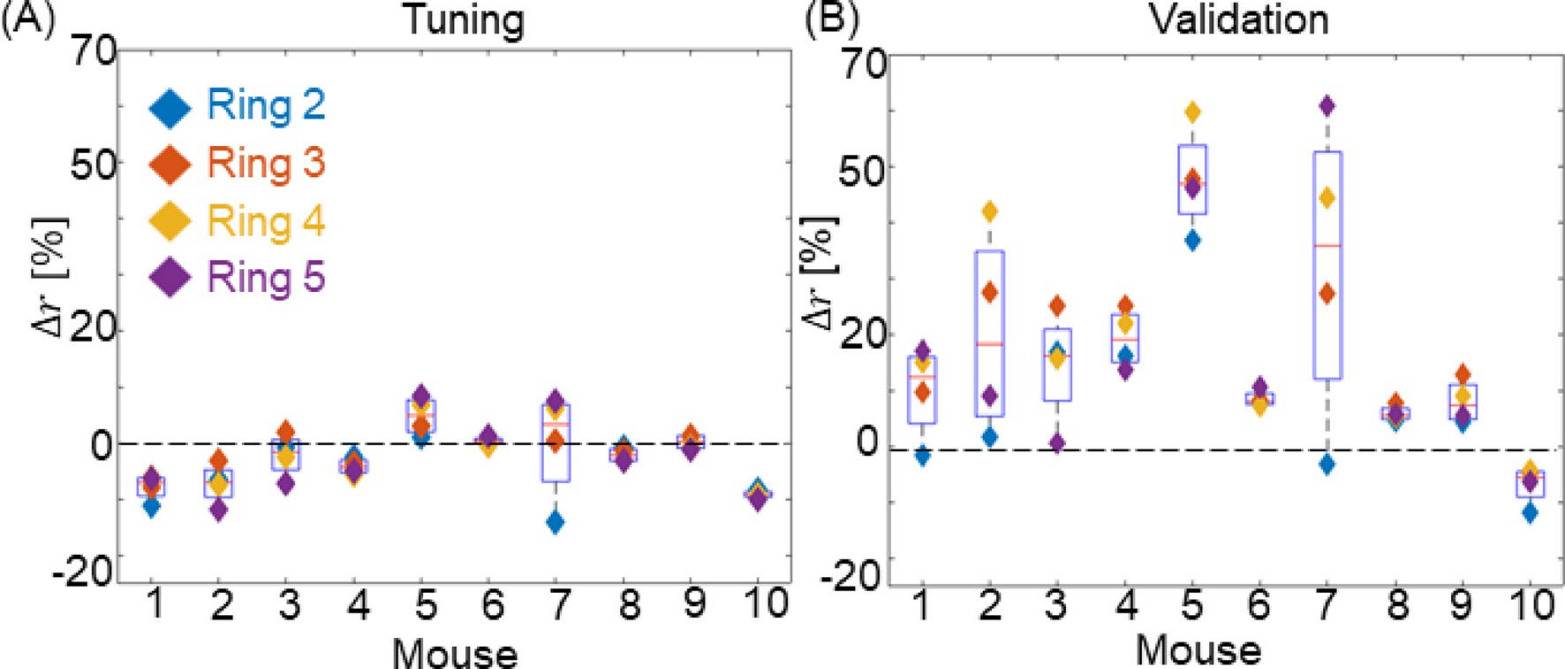
Performance analysis for a single-domain model. **A** Radial expansion error for the tuning (4-month) dataset. **B** Radial expansion error for the validation (6-month) dataset. The tuning dataset captures how well the model can tune the projected 4-month to the observed 4-month growth by adjusting the G&R time constants. The validation dataset used the fitted parameters found in the tuning stage and compared the projected growth to the observed 6-month geometry

Figure 8A shows the radial expansion errors for the tuning stage, comparing the model-calculated measurements to the in vivo-derived measurements for all mice. Positive $\Delta r$ means that the measurements from the predicted geometry overestimated the in vivo measurements. Conversely, negative $\Delta r$ means that the measurements from the predicted geometry underestimated the in vivo measurements. The average results show that the model was effective in capturing the 4-month geometry, with the minimum-magnitude average $\Delta r$ being + 0.26% for mice 7 and 9 and the maximum-magnitude average $\Delta r$ being -9% for mouse 10. The model was within ± 10% for most rings on most mice. Mouse 7, however, despite displaying a small average $\Delta r$ (3.35%), expressed the largest local discrepancy, with $\Delta r$ = − 14% for ring 2, near the inlet and $\Delta r=+7.5 \%$ for ring 4 close to the outlet.

Figure 8B displays the radial expansion error for the validation dataset, in which the parameters derived from the tuning stage were used to predict the 6-month geometry and compared to the 6-month imaging data. Notably, the model's performance varied widely across different mice. The mouse 8 simulations agreed well with the experimental measurement, exhibiting minimal discrepancies across the rings with an average $\Delta r$ = + 5%. Conversely, mice 5 and 7 demonstrated poor model performance. Mouse 5 shows an average $\Delta r$ = + 45% with a moderate discrepancy across the rings, while mice 7 had an average $\Delta r$ = + 32% with a wide range of errors across the rings, indicating that the model was inaccurate in predicting the shape as well as the size. Overall, eight out of ten mice showed $|\Delta r|\le$ 20%, and among those four showed $|\Delta r|\le$ 10%. Although the tuning results of Fig. 8B show relatively little tendency toward overprediction or underprediction (*p* = 0.17), the validation results of Fig. 8B showed a strong tendency for the model to overpredict the growth (*p* < 0.01). To assess the added value of the model over a simple linear projection, we computed the predicted maximum aneurysm radius at 6 months based on the model and also based on a simple linear projection of the 2-month and 4-month values. The model-based prediction of maximum radius had a relative error of 11.3 ± 4% (mean ± SD) over the 10 mice; the linear projection, in contrast, had a relative error of 19.8 ± 20% for the same mice, indicating a less accurate prediction and some cases with very large errors, whereas the detailed model had fewer cases of very large error.

### Single domain model: assessment of performance on axial lengthening

**Fig. 9:**
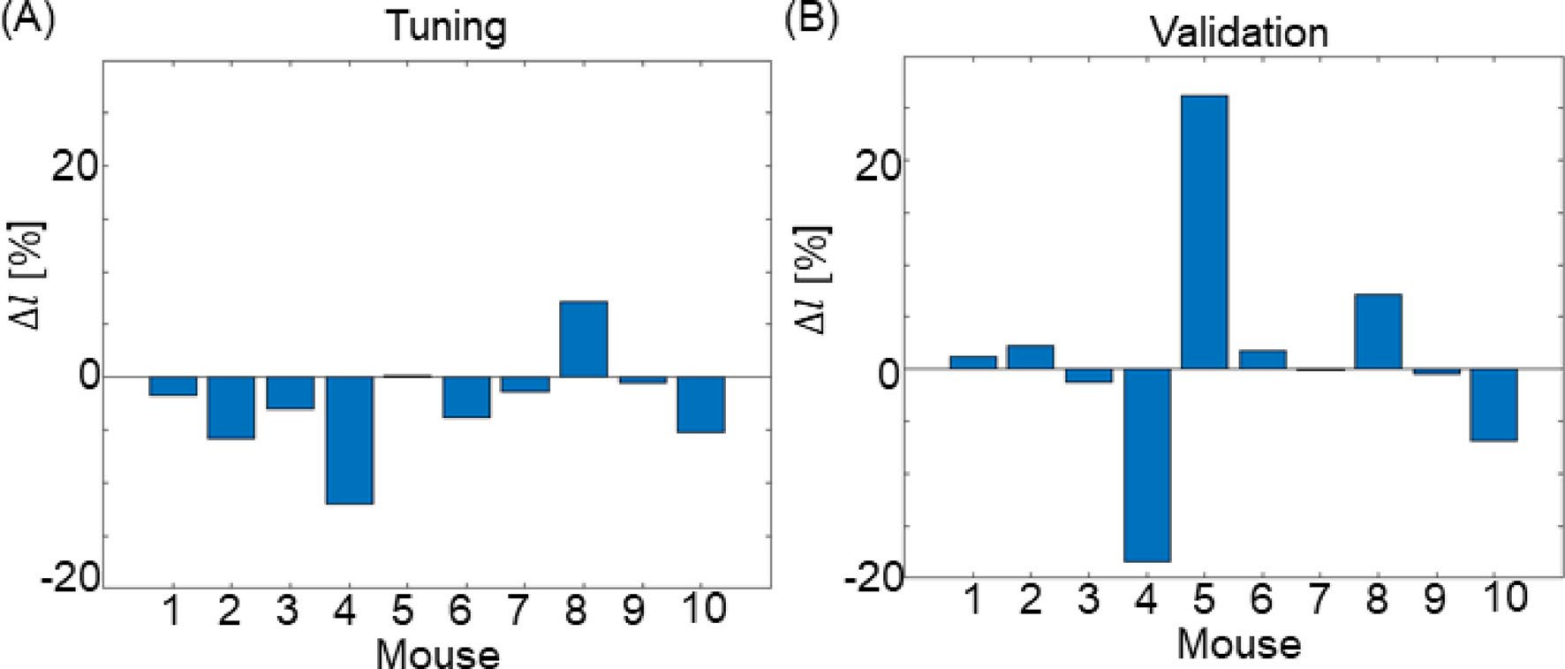
**A** Axial lengthening error for a single-domain model within the tuning dataset. This assessment evaluates the model's ability to fine-tune predicted 4-month axial growth to match observed values by adjusting *the G&R time constants. ****B*** Axial lengthening error for the validation dataset. Employing parameters derived from the tuning stage, this analysis compares predicted axial growth against observed 6-month geometry

Figure 9A shows the axial performance results obtained for the tuning stage. Positive $\Delta l$ means that the predicted geometry measurements overestimated the in vivo measurements, while negative $\Delta l$ denotes underestimation. The axial lengthening error during tuning was comparable to the radial expansion error, with minimum-magnitude $\Delta l$ 0.1% for mouse 5 and maximum-magnitude ${\Delta l}$ being − 12% for mouse 4.

Figure 9B shows the performance analysis for axial lengthening using the validation dataset. As in the analysis of radial expansion, the model's performance displays variability across different mice at this stage. Mouse 7, for which the model had the largest radial expansion error, demonstrates almost no axial lengthening error ($\Delta l$= 0.3%), while mice 4 and 5 exhibit relatively large errors, showing $\Delta l$ = 18%, and $\Delta l$ = 26%, respectively. Overall, eight out ten mice showed $|{\Delta l}|$ < 8%.

### Single domain model: correlation analysis between measured quantities and growth time constant.

**Fig. 10:**
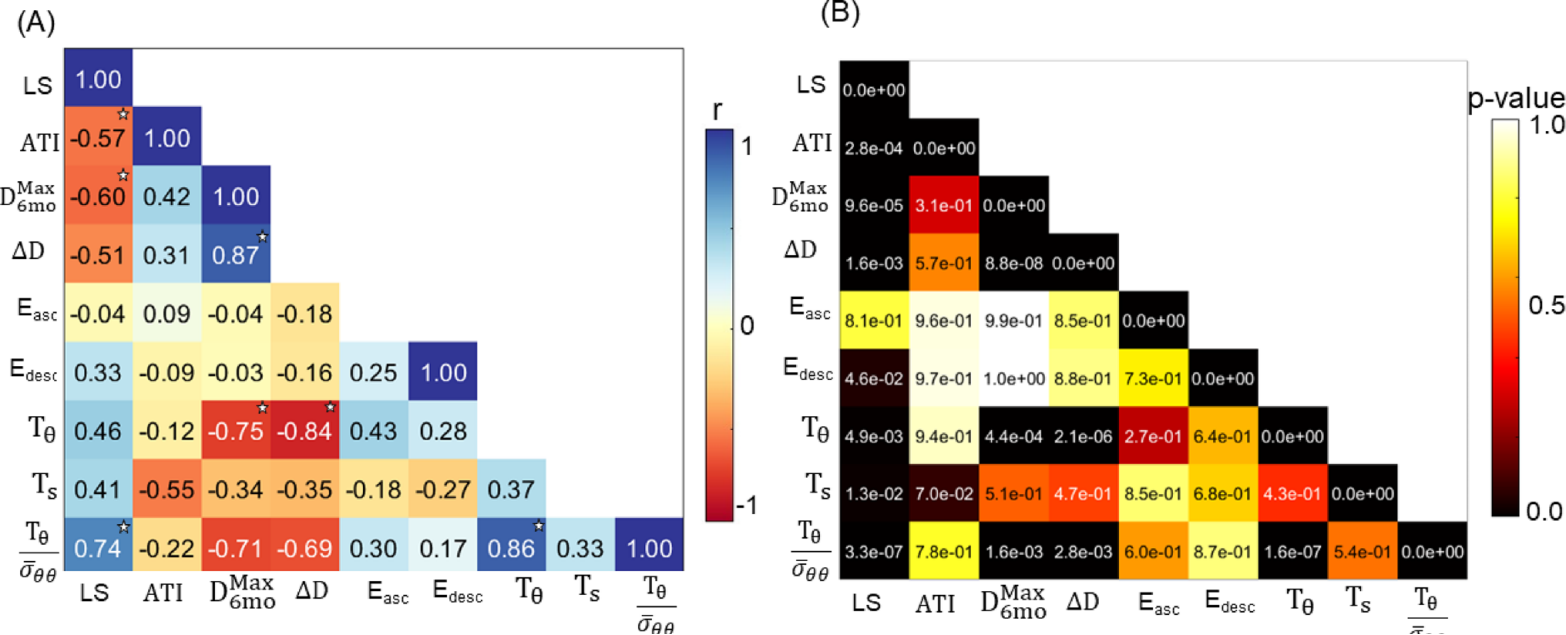
**A** Correlation map between fitted circumferential $({T}_{\theta })$ and axial $({T}_{s})$ growth constant lifespan (LS), aortic tortuosity index (ATI), maximum diameter at 6 months $({D}_{6\text{mo}}^{\text{Max}})$, circumferential growth from 2 to 6mo $(\Delta D)$ and postmortem measurements of ascending and descending Young's modulus, *E*_asc_ and *E*_desc_, respectively. The ratio between the fitted circumferential growth constant and the mean value of the time-averaged ${\bar{\sigma }}_{\theta \theta }$ is incorporated in the analysis. **B** P-values calculated for each one of the correlations. Stars represent the significant correlation at 95% confidence level corrected using Bonferroni correction

Figure 10A shows the correlation map between the computed and measured quantities. The correlation is measured through the Pearson coefficient (*r*) values. Blue color means that the quantities were positively correlated, and red color means that they were negatively correlated.

The strong negative correlation between ${T}_{\theta }$ and both ${D}_{6\text{mo}}^{\text{Max}}$ and $\Delta {\text{D}}$ was expected, considering that the measured circumferential growth was used to tune ${T}_{\theta }$. A modest but significant positive correlation was seen between the ${T}_{\theta }$ and LS (*r* = 0.46, *p* < 0.01). However, a significantly stronger correlation appeared for ${T}_{\theta }$ divided by mean value of the time-averaged $\overline{\sigma }_{\theta \theta }$ (*r* = 0.74, *p* < 1e−7). This ratio, based on the form of Eq. (5) and the fact that the target stress ${\widehat{\sigma }}_{\theta \theta }$ was set to a non-adjustable constant, represents the averaged driving force for tissue growth in the aorta.

Two other pairs showed correlations that, while not significant at the 95% confidence level, were suggestive of avenues for future investigation. ${T}_{\theta }$ showed a very modest correlation with *E*_asc_ (*r* = 0.43, *p* = 0.27), showing a potential association between circumferential growth and remodeling and stiffness of ascending thoracic aorta. On a final note, a moderate negative correlation is observed between ${T}_{s}$ and ATI (*r* = − 0.55, *p* = 0.07), connecting axial growth with geometrical changes along the ascending and especially the descending thoracic aorta.

### Performance of single-domain approach versus multiple-domain approach: mouse 1 analysis

**Fig. 11:**
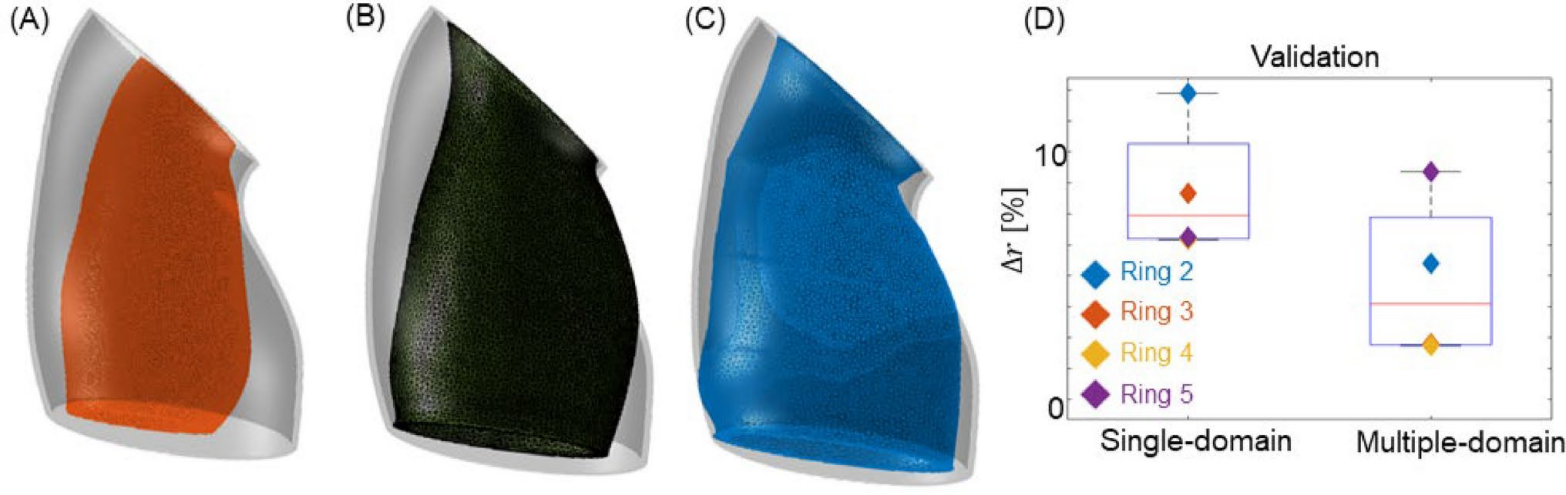
Single-domain versus multi-domain model. **A** 2-month geometry constructed directly from medical scans (orange) and 6-month geometry constructed directly from medical scans (translucent outline). **B** 6-month model-predicted geometry using the single-domain approach (dark green) and 6-month geometry constructed directly from medical scans (translucent outline). **C** 6-month model-predicted geometry using the multiple-domain approach (blue) and 6-month geometry constructed directly from medical scans (translucent outline). **D** Quantitative comparison of the local differences for rings 2 through 5 for single domain versus multiple domain

Figure 11 shows the initial (2-month) geometry and the single-domain and multiple-domain model predictions for the 6-month geometry, with all three cases also showing the actual 6-month geometry as measured by MRI. The use of multiple domains (Fig. 11C) yielded a more accurate prediction than the single-domain approach (Fig. 11B). Figure 11D quantifies the differences between these approaches. There was an approximately 50% reduction in the mean radial error, decreasing from about 7.5% in the single-domain method to roughly 3.75% in the multiple-domain approach. Based on an F-test on the sum of squared errors for nested models (Bonamente 2022) applied to the 4-month data, we calculated a p-value less than 0.001, indicating that the four-domain model is significantly better than the single-domain model even when accounting for the larger number of fitting parameters. We note that statistical significance does not necessarily imply practical significance and that other errors, e.g., in the conversion of the MRI scan to a target geometry, were not considered in the analysis.

## Discussion

This study applied a combination of (1) subject-specific modeling (2) fluid–solid interaction FSI simulations, and (3) a kinematic growth law to maladaptive growth and remodeling of the ascending thoracic aorta in the *Fbln4*^*SMKO*^ mouse. Each one of the three aspects of the simulations was important. The subject-specific models allowed mouse-specific analysis including comparison to lifespan and provided a potential test for future patient-specific studies. The FSI simulations provided accurate estimates of pressure-induced wall stress and wall shear stress. The growth model predicted the time evolution of aortic growth in response to calculated animal-specific stresses.

The validation of the model with uniform growth laws, referred to herein as the single-domain approach, suggested that although the actual growth and remodeling process is extremely complex, much of the observed growth can be predicted by a kinematic growth model with well-parameterized equations. In eight out of ten cases, the computational model produced a radial error of less than 20% when compared to in vivo measurements. We also compared the performance of a multi-domain growth model against the single-domain approach, finding—for the specific mouse model studied here—that the multi-domain model exhibited slightly superior predictive capabilities, offering statistically significantly more accurate estimations than the single-domain approach. This nuanced difference in accuracy suggests the potential benefits of considering domain-specific growth dynamics to capture the spatial heterogeneity of G&R processes (Bersi et al. 2018; Sokolis 2020) but also draws attention to the need for good descriptive data to fit/specify any model.

The primary outcome of our study centered on deriving mouse-specific circumferential and longitudinal growth rate constants as described by Eqs. (5)–(7). Both growth rates showed a modest correlation to the lifespan, with *r* = 0.46 for ${T}_{\theta }$ and *r* = 0.41 for ${T}_{s}$. A larger correlation was seen, however, when we divided the circumferential growth rate constant ${T}_{\theta }$ against the time-averaged circumferential stress ${\sigma }_{\theta \theta }$ (representing the net driving force for growth). It is perhaps not surprising that growth is driven by a combination of the tissue’s mechanical environment (${\sigma }_{\theta \theta }$) and its intrinsic nature (${T}_{\theta }$), but, notably, the strongest correlation was seen when both factors were considered.

A moderate correlation was observed between ${T}_{s}$ and ATI (*r* = − 0.55, *p* = 0.07), hinting at a potential association between axial growth and unhealthy geometrical changes along the descending thoracic aorta (Humphrey et al. 2009). This effect was of relatively low significance, but, intriguingly, it relates the rate constant calculated for the ascending aorta with the observed behavior of the descending aorta. The observation (Franken et al. 2015) that aortic tortuosity correlates with ATAA severity in Marfan patients further suggests that axial growth merits further consideration.

A positive strong correlation between ${T}_{\theta }$ and both ${D}_{6\text{mo}}^{\text{Max}}$ and $\Delta {\text{D}}$ was calculated, underscoring their relationship with circumferential growth. The correlation between ${T}_{\theta }$ and ${T}_{s}$ was quite weak, indicating potential independence or minimal association between these factors.The computational model successfully captured the overall growth of the aorta in the *Fbln4*^*SMKO*^ mouse and provided insights into the local growth patterns. However, some limitations need to be addressed for the model to be more widely applicable. Currently, the model primarily considers only hemodynamic factors, yet substantial evidence points to the critical role of biochemical factors such as inflammatory markers and matrix metalloproteinases in growth and remodeling (Hadler-Olsen et al. 2011). In diseased tissues, such as those in aneurysmal patients, there is a noticeable imbalance between the rates of matrix production and degradation, as well as cell phenotypes. This imbalance leads to deviations from normal, mechano-regulated tissue properties, which a stress-based model—without the incorporation of cell-mediated chemical signaling—fails to capture effectively (Irons and Humphrey 2020; Schwartz et al. 2018). Incorporating these elements into the model framework could significantly heighten its predictive accuracy. Another limitation is the simplified assumption of homogeneous and isotropic material properties in the aortic tissue, disregarding its inherently anisotropic and heterogeneous nature (Bersi et al. 2018; Holzapfel and Weizsäcker 1998; Bellini et al. 2017; Farzaneh et al. 2019). A more mechanically accurate model would be appealing in terms of understanding the tissue behavior. However, it would also increase the number of model parameters and could contribute to an overfitting problem. Future research should aim for more sophisticated material models to accurately represent the mechanical complexity and the concurrent development of imaging/instrumentation methods that could provide additional information for testing the model. For example, if tissue stiffness were measured in vivo, [e.g. Davis et al. (2016)], it could provide an important additional test of growth model. Additionally, in vivo pressure measurement, as recently used in pulmonary remodeling in response to stenosis (Kozitza et al. 2024), could also serve as an important validation tool.

Another key limitation of this study is the assumption of uniform wall thickness, while in reality, wall thickness varies, especially in aneurysmal tissue. This simplification helped reduce model complexity but may have affected the accuracy of the predicted growth and remodeling (G&R) behavior. Additionally, thrombus formation, which alters wall stress and supports the vessel wall, was not modeled. These factors could have influenced the G&R process in our simulations. Future models should incorporate variable wall thickness and thrombus formation, using advanced imaging techniques like high-resolution MRI or 3D ultrasound for more precise predictions of aneurysm progression. Additionally, the assumption of non-animal-specific flow boundary conditions could impact the accuracy of stress distributions, as boundary conditions derived from generalized or literature-based values do not fully capture the physiological variability of individual subjects. Future work should aim to incorporate boundary conditions tailored to each animal to enhance the precision of the model.

The study also focused on a relatively small number of mice. While the results are encouraging, validating the model with a larger dataset is essential to confirm its generalizability. Additionally, investigating the model's performance across different mouse models of ATAA would further strengthen its applicability and perhaps provide insights into how different genetic challenges drive aneurysm formation and growth.

There have been a number of different attempts in recent years to describe aortic growth and remodeling using computational models. This study’s approach combines subject-specific fluid–solid interaction (FSI) simulations with kinematic growth and remodeling (G&R) laws to model the ascending thoracic aorta (ATAA) in the Fbln4^SMKO^ mouse, but it does not account for detailed structure and mechanics, nor does it account for the underlying biological processes. Humphrey and co-workers have notably developed highly sophisticated models for aortic growth and remodeling using coupled intracellular signaling and tissue-level changes (e.g., Estrada and Humphrey 2025; Estrada et al. 2024.) but have used idealistic geometries, as did the extremely structurally detailed model of Dalbosco et al. (2023). Other studies (Jamaleddin Mousavi, et al. 2021; Laubrie et al. 2022) are similar in approach to ours but have focused on human data, using more sophisticated but generically parameterized material models for the aortic wall and a smaller subject population size. Finally, a hybrid physical-deep-learning model has shown promise in predicting abdominal aortic aneurysm growth (Jiang et al. 2020). Thus, it must be concluded that, at present, there is no clear best way to predict aortic aneurysm growth and remodeling and that the integration of multiple approaches—with new ones as yet unexplored—will eventually lead to improved patient care.

While this study focuses on modeling aortic growth in the Fbln4^SMKO^ mouse model, the methodology has significant potential for translation to human applications. The integration of subject-specific FSI simulations with G&R models could be adapted to patient-specific geometries derived from clinical imaging, such as CT or MRI scans. By using personalized boundary conditions and mechanical properties obtained from non-invasive measurements or prior studies, the framework could predict individual aortic growth trajectories. In human subjects, such modeling could aid in assessing the progression of aortic aneurysms or other vascular pathologies. For instance, correlations between growth parameters and risk factors, such as wall stress or mechanical properties, could inform surgical decision-making. Although this methodology has potential for human applications, several challenges remain. Patient-specific data, such as mechanical properties of the aorta and accurate boundary conditions is not often readily available, the non-invasive techniques to obtain this data can be expensive or lack the necessary accuracy. Furthermore, human variability in age, sex, and comorbidities complicates generalization from mouse studies, necessitating robust parameter estimation techniques and larger datasets for clinical relevance.

## Conclusion

This study presents a comprehensive approach to understanding maladaptive aortic growth in the Fbln4^SMKO^ mouse model using a combination of subject-specific modeling, fluid–structure interaction (FSI) simulations, and a kinematic growth and remodeling (G&R) model. The results demonstrate that even a simplified G&R model can capture significant aspects of the growth patterns in the ascending thoracic aorta, with radial and axial growth predictions showing a strong correlation to biomechanical and geometric metrics. Furthermore, the enhanced predictive capability of the multi-domain model underscores the importance of incorporating spatial heterogeneity in modeling G&R processes.

Notably, the correlation between circumferential growth parameters and lifespan, as well as the relationship between axial growth and aortic tortuosity, highlights the potential of computational models to predict aneurysm progression and associated risks. However, limitations such as the assumption of uniform wall thickness and the exclusion of biochemical influences suggest that future studies should aim to integrate these factors for a more accurate representation of the G&R process.

## Supplementary Information

Below is the link to the electronic supplementary material.

Supplementary file1 (DOCX 72 kb)

## Data Availability

No datasets were generated or analyzed during the current study.
