# Supplementary material for: Ascending aortic aneurysm growth in the Fbln4SMKO mouse is consistent with uniform growth laws
Source: Biomech Model Mechanobiol. 2025 Jul 21;24(5):1485–99. doi: 10.1007/s10237-025-01972-5 (PMC12454605; doi:10.1007/s10237-025-01972-5)
Supplement: Supplementary file 1 — Supplementary file1 (DOCX 72 kb) [file 10237_2025_1972_MOESM1_ESM.docx]

## **Supplemental information**

The analysis for the sensitivity associated with the number of rings is shown Figure . In Figure A we can see that it takes the same number of iterations for all the different numbers of rings to reach convergence. And they converge to the same results as shown in Figure B for the circumferential time constant $T_{\theta}$, and Figure C for the axial time constant, $T_{s}$. The initial guess for each constant was 70 days.


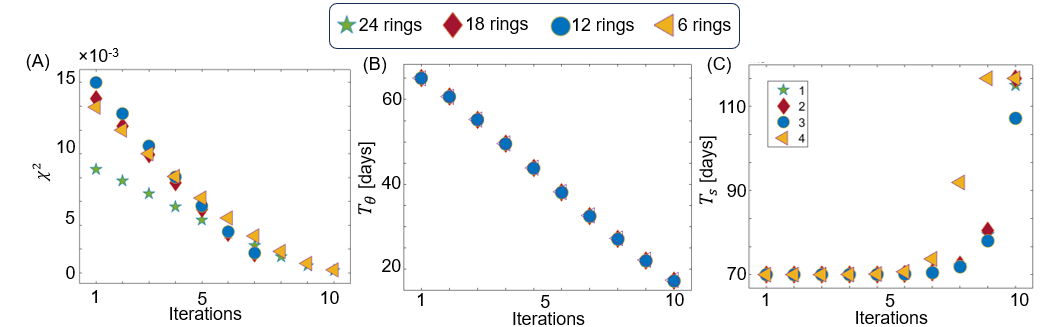


**Figure S1:** (A) Analysis of convergence using $\chi^{2}$for different number of rings. (B) Convergence in the time constants associated to circumferential growth, $T_{\theta}$ and (C) axial growth, $T_{s}$. Difference marker shows analysis of sensitivity of the model to the number of rings used for the optimization.
